# Construction and verification of risk prediction model of osteoporotic fractures in patients with osteoporosis in China

**DOI:** 10.3389/fpubh.2024.1380218

**Published:** 2024-03-21

**Authors:** Peifang Xia, Yingqing Jiang, Feng Cai, Shuzhi Peng, Zhouya Xu

**Affiliations:** ^1^Department of Orthopedic Surgery, The First Affiliated Hospital of Soochow University, Suzhou, Jiangsu, China; ^2^Graduate School, Shanghai University of Traditional Chinese Medicine, Shanghai, China

**Keywords:** osteoporosis, OPF, prediction model, retrospective cohort study, China

## Abstract

**Objective:**

To explore the influencing factors of osteoporotic fractures (OPF) in patients with osteoporosis, construct a prediction model, and verify the model internally and externally, so as to provide reference for early screening and intervention of OPF in patients with osteoporosis.

**Methods:**

Osteoporosis patients in the First Affiliated Hospital of Soochow University were selected, and the medical records of patients were consulted through the Hospital Information System (HIS) and the data management platform of osteoporosis patients, so as to screen patients who met the criteria for admission and discharge and collect data. SPSS 26.0 software was used for single factor analysis to screen statistically significant variables (*p* < 0.05). The influencing factors of OPF were determined by multivariate analysis, and a binary Logistic regression model was established according to the results of multivariate analysis. Hosmer-Lemeshow (H-L) goodness of fit and receiver operating characteristic curve (ROC) were used to test the model’s efficiency, and Stata 16.0 software was used to verify the Bootstrap model, draw the model calibration curve, clinical applicability curve and nomogram.

**Results:**

In this study, the data of modeling set and verification set were 1,435 and 580, respectively. There were 493 (34.4%) cases with OPF and 942 (65.6%) cases without OPF in the modeling set. There were 204 (35.2%) cases with OPF and 376 (64.8%) cases without OPF. The variables with statistically significant differences in univariate analysis are Age, BMI, History of falls, Usage of glucocorticoid, ALP, Serum Calcium, BMD of lumbar, BMD of feminist neck, *T* value of feminist neck, BMD of total hip and *T* value of total hip. The area under ROC curve of the risk prediction model constructed this time is 0.817 [95%CI (0.794 ~ 0.839)], which shows that the model has a good discrimination in predicting the occurrence of OPF. The optimal threshold of the model is 0.373, the specificity is 0.741, the sensitivity is 0.746, and the AUC values of the modeling set and the verification set are 0.8165 and 0.8646, respectively. The results of Hosmer and Lemeshow test are modeling set: (χ^2^ = 6.551, *p* = 0.586); validation set: [(χ^2^ = 8.075, *p* = 0.426)]. The calibration curve of the model shows that the reference line of the fitted curve and the calibration curve is highly coincident, and the model has a good calibration degree for predicting the occurrence of fractures. The net benefit value of the risk model of osteoporosis patients complicated with OPF is high, which shows that the model is effective.

**Conclusion:**

In this study, a OPF risk prediction model is established and its prediction efficiency is verified, which can help identify the high fracture risk subgroup of osteoporosis patients in order to choose stronger intervention measures and management.

## Introduction

Osteoporosis (OP) is a kind of systemic bone disease characterized by low bone mass and damage of bone microstructure, which leads to increased bone brittleness and prone to fracture (1). Osteoporotic fractures (OPF) are low-energy or non-violent fractures, which refer to fractures without obvious external force in daily life, also known as brittle fracture (2). According to a set of data published by the International Osteoporosis Foundation, 33% of women and 20% of men in the world will suffer an OPF after the age of 50 (3). An epidemiological survey of OP in China shows that the prevalence rate of OPF in people over 50 years old is 26.6% (4). The cost of OPF in China has reached nearly 65 billion yuan (5). It is estimated that the number of osteoporotic fractures in China will be 4.83 million in 2035 and 5.99 million in 2050, and the related medical expenses will be 25.43 billion dollars, which will bring huge economic burden to individuals, families and society (6, 7). Therefore, it is of great significance for the prevention and treatment of OPF to evaluate the fracture risk of OP population and find the high-risk population.

Bone mineral density (BMD) measured by Dual X-ray absorptiometry (DXA) is the gold standard for diagnosing OP, and an important index for judging the fracture risk of OP patients in clinic (8). However, some studies have found that the overlapping degree of BMD values between patients with fracture and patients without fracture is as high as 45% (9), indicating that the BMD level of patients with osteoporosis is not completely parallel to the probability of fracture. This suggests that BMD is not the only determinant of the risk of OPF in patients with OP. Therefore, relying only on DXA may miss the high-risk fracture population in OP patients and miss the opportunity of intervention.

Some studies have pointed out that age, BMI and lifestyle are independent risk factors of OPF, and on this basis, a number of different fracture risk assessment tools have been developed, among which fracture risk assessment tool (FRAX) is the most widely used (10, 11). However, the people in the FRAX development queue mainly come from the United States, Britain, Australia and other countries, which cannot fit well with people from different countries with different ethnic, regional and cultural backgrounds. Studies have shown that the FRAX score underestimates the fracture risk of women in China (12), which may be related to the fact that FRAX does not include some risk factors that have great influence in China population. At present, there have been a lot of studies on the influencing factors of OPF, but the targeted discussion on the people who have been diagnosed with osteoporosis is insufficient, and there is also a lack of evaluation tools that meet the characteristics of China population (13).

OP has become one of the important diseases that threaten the life and health of middle-aged and older adult people, and the number of patients is increasing year by year. The most effective way to deal with OP is early detection, but the shortage of experts and instruments, high examination cost and instrument radiation seriously restrict the early diagnosis of OP. Therefore, it is urgent to build a convenient and accurate risk prediction model for screening and early diagnosis of OP economically and efficiently.

In this study, the influencing factors of OPF in patients with OP in China were identified by retrospective analysis, and a risk prediction model was constructed and its clinical efficacy was verified. Our OPF risk prediction model, as a non-invasive screening method, contains many factors such as clinical risks and lifestyle, and it is simple and easy to use, and has passed external verification, so it can be popularized and applied in clinic, providing reference for early screening and intervention of OPF in OP patients.

## Materials

### Data source

Through the hospital information system (HIS) and the data management platform of osteoporosis patients, the patients with osteoporosis who meet the admission criteria were screened, and the missing data were supplemented by telephone follow-up or face-to-face inquiry. From January 2020 to June 2023, 1,500 patients with osteoporosis in the First Affiliated Hospital of Soochow University were selected, and 1,045 patients were finally included as the data of the modeling set. The data in the validation set came from a 3A hospital in Shanghai, and 600 patients with osteoporosis were screened out, and finally 580 patients were included.

### Patient selection

Inclusion criteria: (1) The age is between 40 to 90 years; (2) Meet the WHO diagnostic criteria for osteoporosis; and (3) Not treated with anti-osteoporosis drugs. Exclusion criteria: (1) Secondary osteoporosis or other metabolic bone diseases; (2) Violent fracture (such as car accident, falling from a height, etc.); (3) Pathological fracture caused by tumor bone metastasis; (4) Other diseases affecting bone or soft tissue metabolism, such as type I diabetes, hyperthyroidism, bone tuberculosis, etc.; (5) History of malignant tumor; (6) Severe cardiopulmonary disease or hepatic and renal insufficiency; and (7) Personal data and data are incomplete.

### Determination of osteoporotic fractures

According to the WHO diagnostic criteria (14), it refers to the fracture that occurs without obvious external force or external force that does not cause fracture in daily life. In this study, the diagnosis of OPF mainly depends on clinical imaging, and the patient’s self-reported history of OPF needs clinical records or imaging support. Fracture sites include main fracture sites: vertebrae, hips, pelvis, proximal humerus and distal forearm.

## Methods

### Predictor variables

The basic information of patients includes age, gender, BMI, patients’ fall history in the past year, parents’ hip fracture history, past medical history, exercise, sunshine, dairy products intake, smoking, drinking, usage of glucocorticoid and other basic information. Laboratory indexes include: Alkaline phosphatase (ALP), creatinine, serum calcium, blood phosphorus, C-terminal peptide of type I collagen (CTX), N-terminal peptide of type I collagen (NTX), vitamin D3, lumbar bone density, total hip bone density and femoral neck bone density.

### Calculation of sample size

There are 33 risk factors involved in this study. According to EPP principle (15), each risk factor in the modeling set needs 5–10 positive patients. According to the epidemiological survey of osteoporosis in China, the incidence of fracture in patients with osteoporosis is 26.6% (16). Considering the loss rate of 20%, the sample size required in this study is at least: *n* = (33*5/0.266)*1.2 = 744. It has been pointed out in some literatures that the sample size for external verification of prediction model should be at least 100 cases (17), and then considering the loss rate of 20%, the sample size for verification set in this study should be at least 125 cases. For the prediction model, the larger the sample size, the more generalizable the prediction model is. According to this survey, the sample size we can obtain the data of the modeling set and the verification set are 1,435 and 580, respectively.

### Data analysis

The collected data is entered and sorted by EpiData 3.1 software. All the data are entered and checked by two people. If there are any differences between the two people, the third person will check the data. SPSS 26.0 and Stata 16.0 were used for statistical analysis and drawing receiver operating characteristic curve (ROC), calibration curve, Decision Curve Analysis (DCA) and nomogram. Counting data are described by frequency and percentage. Univariate logistic regression method was used to evaluate the characteristic differences between fracture group and non-fracture group, and the variables with *p*-values<0.05 in univariate analysis were introduced into multivariate logistic regression as independent predictors. We estimated the correlation strength between fracture risk and predictors in patients with osteoporosis through HR and 95%CI. The significant variables in multivariate Logistic regression analysis are used to construct prediction models and draw nomograms. The Bootstrap method was strengthened and the samples were re-sampled for 1,000 times for internal verification.

## Results

### Descriptive analyses

In this study, the data of modeling set and verification set were 1,435 and 580, respectively. There were 493 (34.4%) cases with OPF and 942 (65.6%) cases without OPF in the modeling set. There were 204 (35.2%) cases with OPF and 376 (64.8%) cases without OPF. The demographic information of the two data sets is shown in Table 1.

**Table 1:** Demographic information [*n* (%)].

Variables	Modeling set		χ^2^(*p*)	Verification set		χ^2^(*p*)
	NO-OPF	OPF		NO-OPF	OPF	
Gender						
Female	276 (19.2)	132 (9.2)	1.014 (0.325)	265 (45.7)	148 (25.5)	0.276 (0.632)
Male	666 (46.4)	361 (25.2)		111 (19.1)	56 (9.7)	
Age						
<60 years	527 (36.7)	190 (13.2)	39,216 (<0.01)	201 (34.7)	80 (13.8)	10.740 (<0.01)
≥60 years	415 (28.9)	303 (21.1)		175 (30.2)	124 (21.3)	
Marital status						
Married	569 (39.7)	301 (21.0)	0.058 (0.820)	221 (38.2)	118 (20.3)	0.047 (0.860)
Unmarried	373 (26.0)	192 (13.4)		155 (26.7)	86 (14.8)	
Length of education						
<6 years	460 (32.1)	233 (16.2)	0.320 (0.579)	168 (29.0)	94 (16.2)	0.104 (0.793)
≥6 years	482 (33.6)	260 (18.1)		208 (35.9)	110 (18.9)	

### Construction of risk prediction model

**Figure 1:**
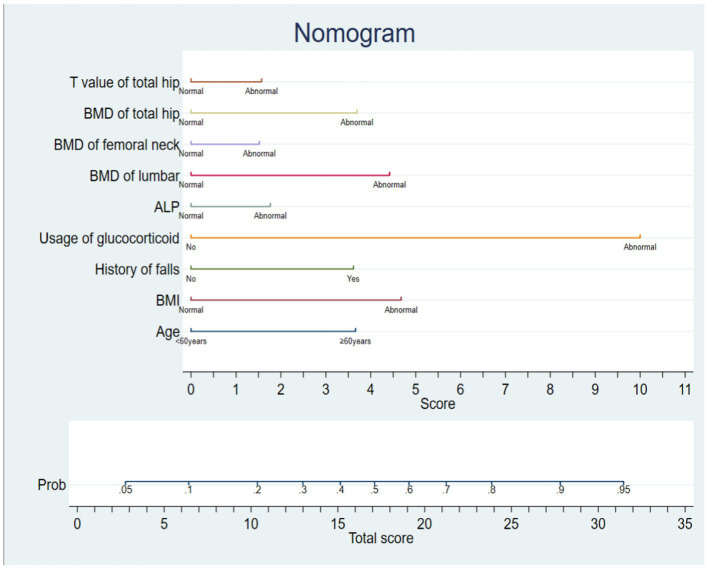
Nomogram of risk for OPF.

The risk factors in modeling set and verification set were analyzed by single factor. The variables with statistically significant differences (*p* < 0.05) in univariate analysis were analyzed by multivariate analysis, as shown in Table 2. According to the results of multivariate analysis, the variables with statistically significant differences (*p* < 0.05) in multivariate analysis are used to construct the risk prediction model of OPF. The nomogram model of OPF risk is determined by stata 16.0 software, and the scores of independent influencing factors are determined according to the clinical data of patients, and the sum corresponding to the prediction probability is the fracture risk of patients. See Figure 1.

**Table 2:** Results of logistic regression analysis.

Variables	Modeling set OR(95%CI)		Verification set OR(95%CI)	
	Univariate analysis	Multivariate analysis	Univariate analysis	Multivariate analysis
Age	2.025 (1.621 ~ 2.529)**	2.121 (1.634 ~ 2.753)**	1.78 (1.259 ~ 2.518)*	1.562 (0.95 ~ 2.567)*
BMI	2.48 (1.98 ~ 3.106)**	2.613 (2.005 ~ 3.406)**	2.129 (1.504 ~ 3.013)**	2.138 (1.301 ~ 3.512)*
History of falls	2.015 (1.615 ~ 2.513)**	2.1 (1.618 ~ 2.726)**	1.865 (1.32 ~ 2.635)**	1.750 (1.061 ~ 2.887)*
Usage of glucocorticoid	7.795 (5.962 ~ 10.192)**	7.791 (5.845 ~ 10.385)**	3.489 (2.443 ~ 4.982)**	3.100 (1.872 ~ 5.131)**
ALP	1.403 (1.093 ~ 1.801)*	1.437 (1.066 ~ 1.937)*	5.802 (3.986 ~ 8.443)**	6.376 (3.814 ~ 10.66)**
Serum Calcium	1.394 (1.036 ~ 1.876)*	(~)	6.649 (4.435 ~ 9.968)**	9.858 (5.526 ~ 17.586)**
BMD of lumbar	2.626 (1.931 ~ 3.572)**	2.478 (1.711 ~ 3.59)**	2.088 (1.266 ~ 3.444)*	5.517 (3.272 ~ 9.302)**
BMD of femoral neck	1.414 (1.086 ~ 1.842)*	1.366 (0.999 ~ 1.868)*	5.348 (3.667 ~ 7.8)**	(~)
*T* value of femoral neck	1.304 (1.018 ~ 1.671)*	(~)	(~)	(~)
BMD of total hip	1.957 (1.45 ~ 2.64)**	2.134 (1.478 ~ 3.082)**	5.022 (3.313 ~ 7.611)**	3.491 (1.934 ~ 6.303)**
*T* value of total hip	1.362 (1.032 ~ 1.798)*	1.38 (0.979 ~ 1.947)*	4.158 (2.813 ~ 6.145)**	3.244 (1.87 ~ 5.63)**

**, *p* < 0.01; *, *p* < 0.05.

### Distinguishing degree of model

**Figure 2:**
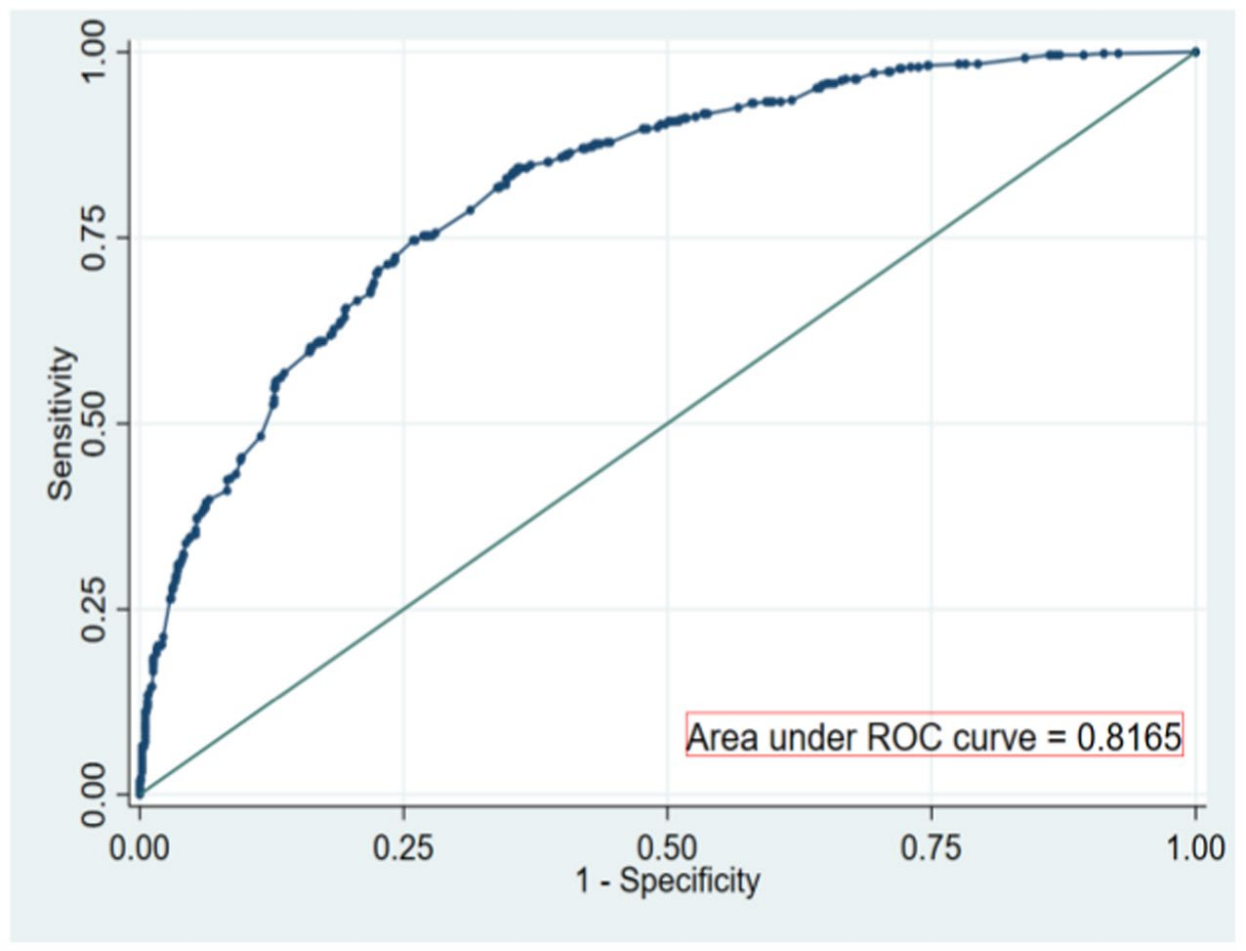
ROC curve for modeling set.

**Figure 3:**
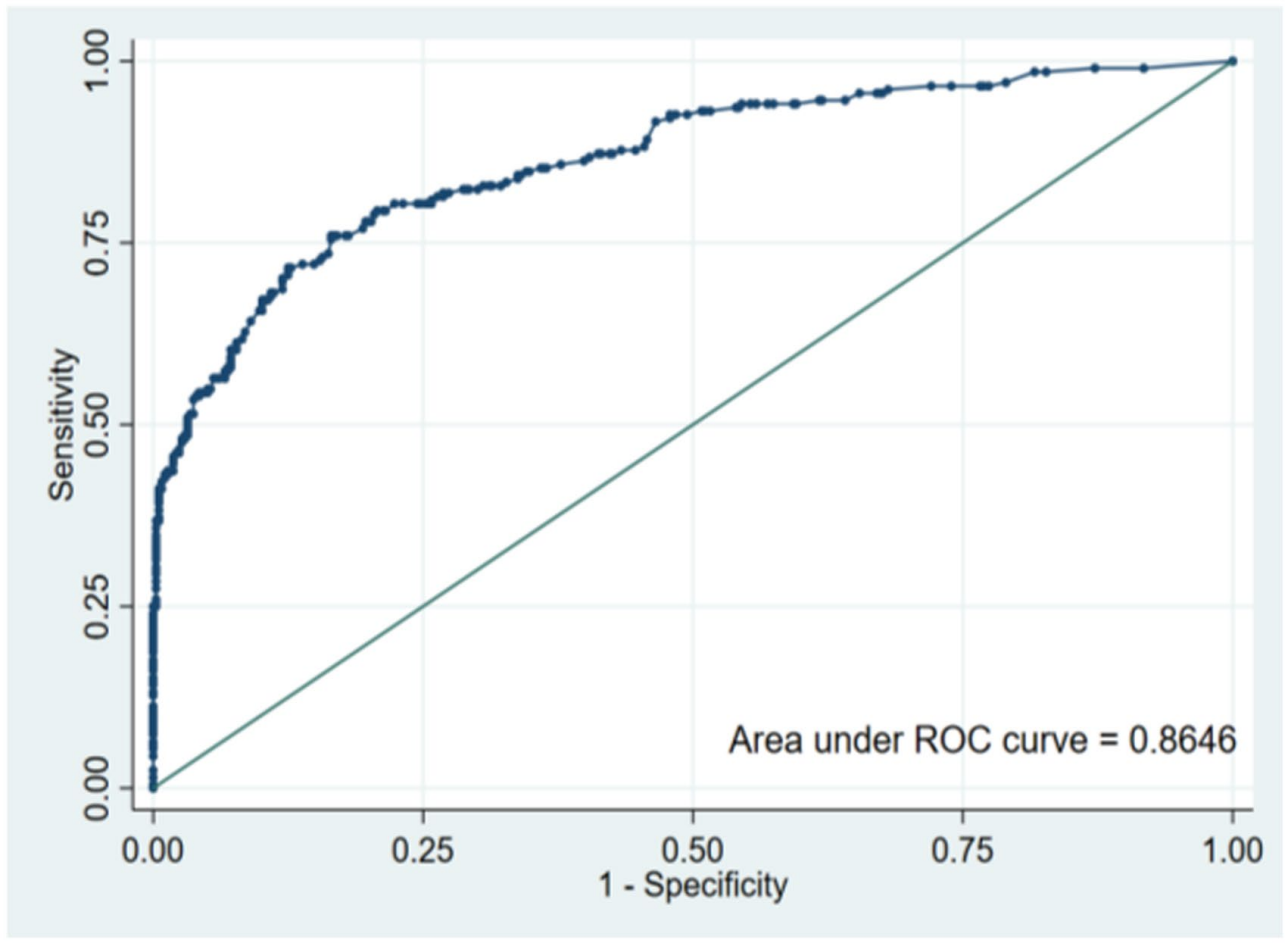
ROC curve for validation set.

The area under ROC curve of the risk prediction model constructed this time is 0.817 [95%CI (0.794 ~ 0.839)], which shows that the model has a good discrimination in predicting the occurrence of OPF. The optimal threshold of the model is 0.373, the specificity is 0.741, the sensitivity is 0.746, and the AUC values of the modeling set and the verification set are, respectively, 0.8165 and 0.8646, as shown in Figures 2, 3.

### Calibration degree of model

**Figure 4:**
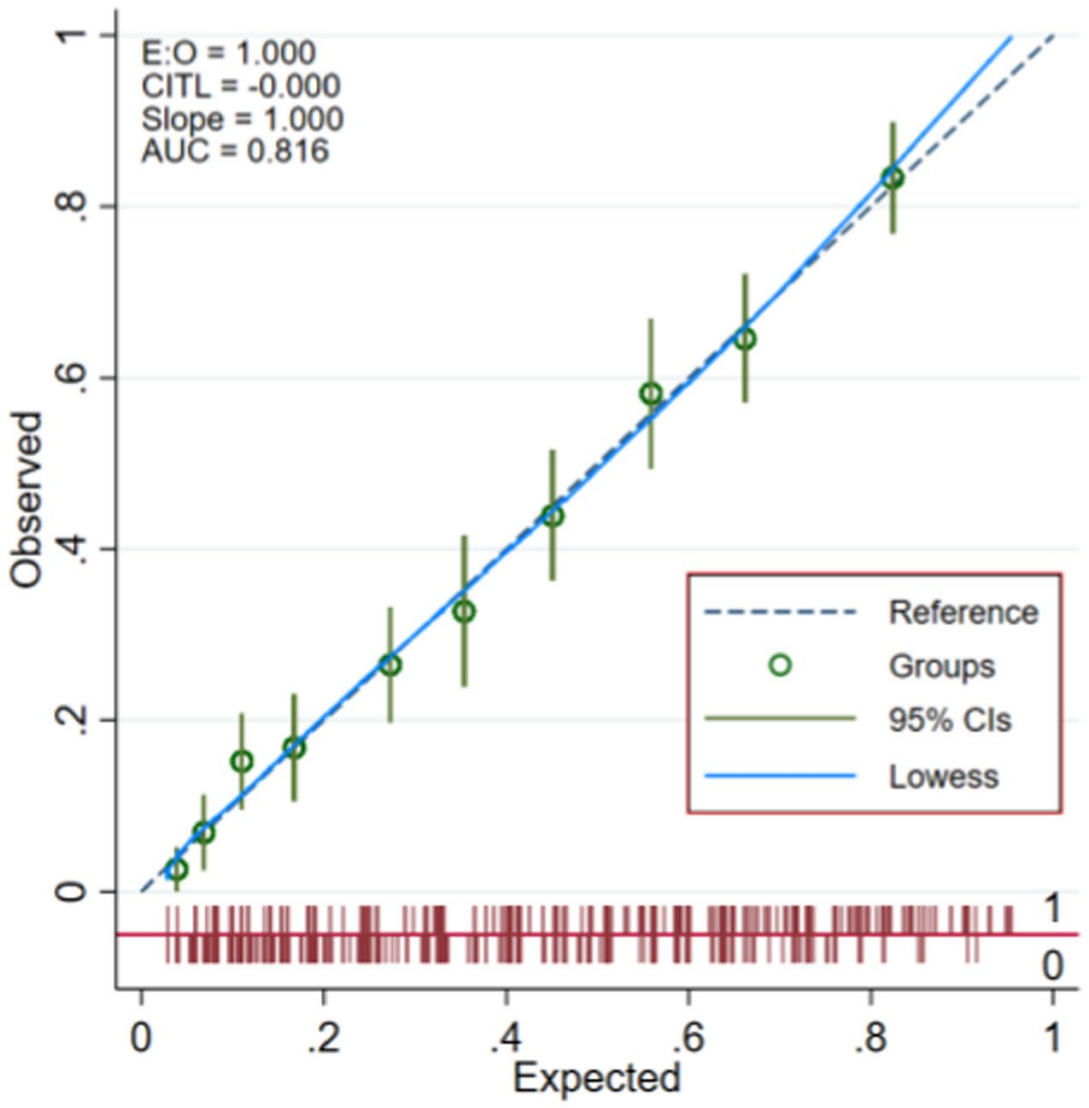
Modeling set calibration curve.

**Figure 5:**
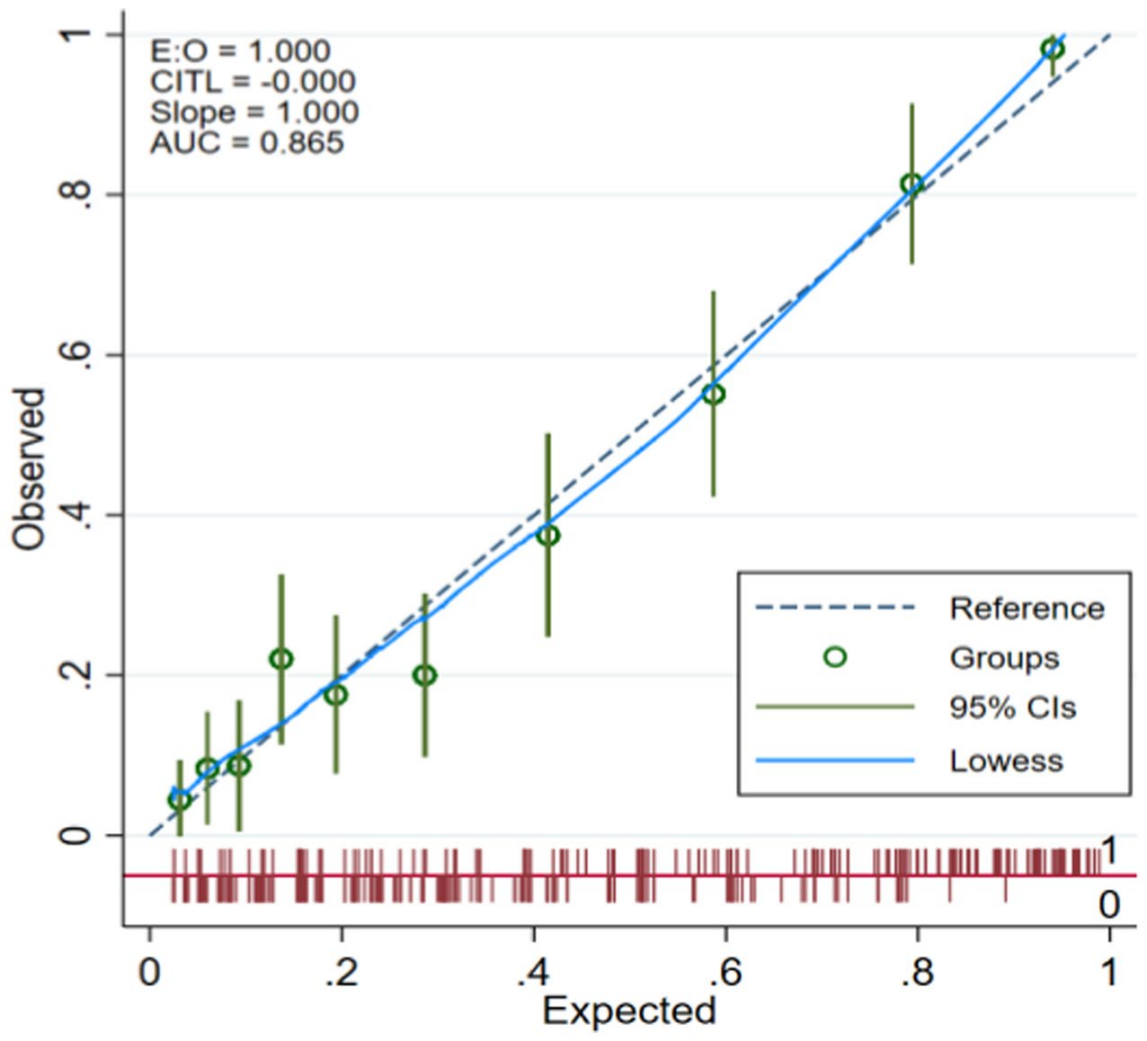
Verification set calibration curve.

The model is verified internally by Bootstrap method, and the original data is sampled 1,000 times, and the original data is repeatedly sampled for 1,000 times. The results of Hosmer and Lemeshow test are, respectively, modeling set: (χ^2^ = 6.551, *p* = 0.586) and validation set: (χ^2^ = 8.075, *p* = 0.426). Drawing the calibration curve of the model shows that the fitted curve has a high degree of coincidence with the 45 reference line of the calibration curve, and the model has a good calibration degree for predicting the occurrence of fractures. The calibration curves are shown in Figures 4, 5.

### Clinical application

**Figure 6:**
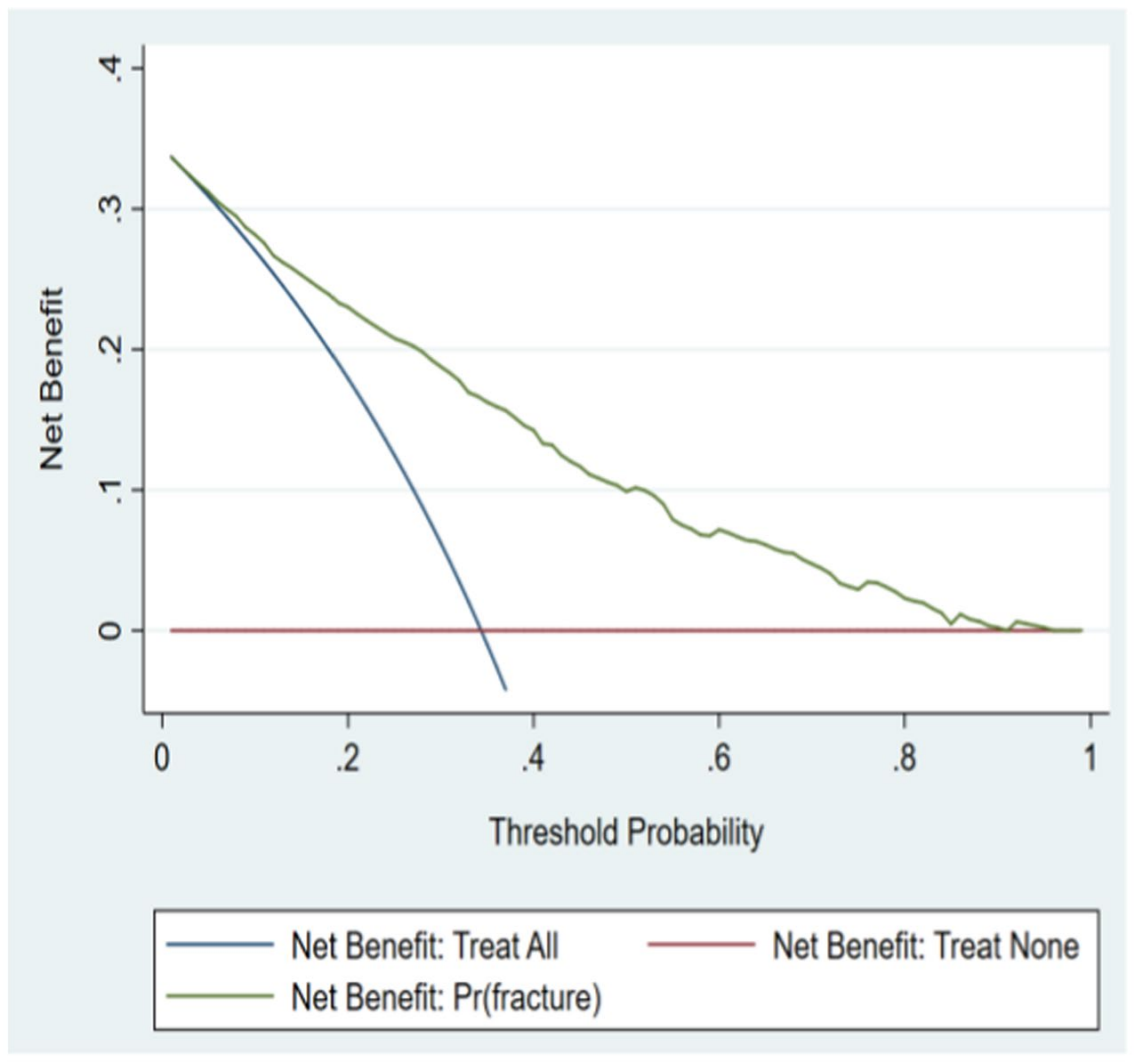
Decision curve of modeling set.

**Figure 7:**
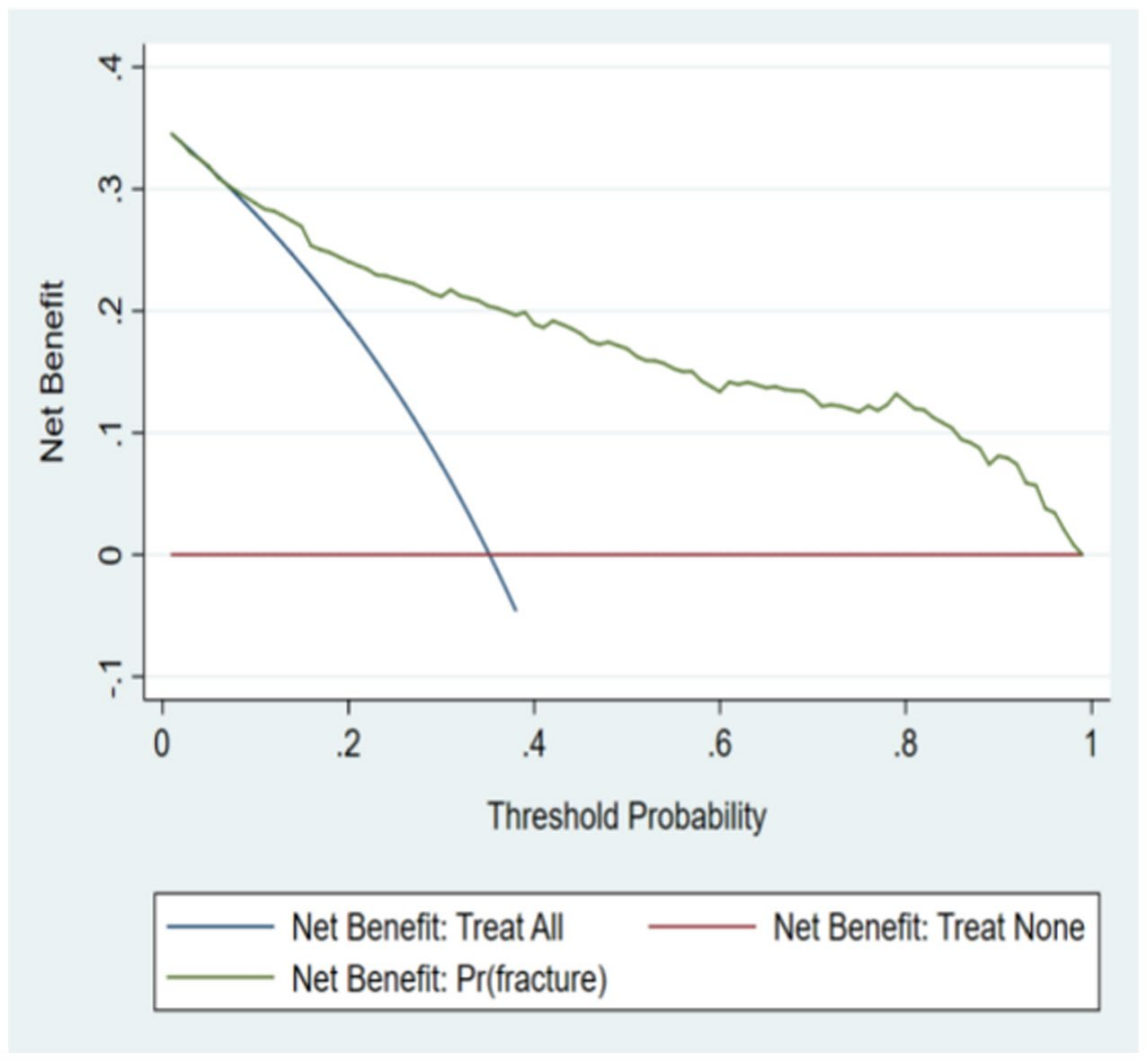
Decision curve of verification set.

From the clinical decision curve, it can be seen that the net benefit value of the risk model of osteoporosis patients complicated with OPF is high, which shows that the model is effective. See Figures 6, 7 for details.

## Discussion

This study systematically compares the differences in general demographic information, bone mineral density, experimental indicators and lifestyle between patients with osteoporosis and those without fracture. The results show that age, BMI, history of falls, usage of glucocorticoid, ALP, serum calcium, BMD of lumbar, BMD of femoral neck, *T* value of femoral neck, BMD of total hip and *T* value of total hip are independent influencing factors of OPF. Among these risk factors, the unchangeable factors are age and fall history, and the changeable factors are BMI, ALP, Serum Calcium and bone mineral density. This study found that the OR value of Usage of glucocorticoid and OPF was the highest (OR = 7.795, 95%CI: 5.845–10.385). A study in Japan pointed out that the probability of fracture was 23.7% and the risk of death was higher in patients treated with high dose of glucocorticoid (18). Glucocorticoid not only directly inhibits osteogenesis by promoting osteoblast and osteocyte apoptosis, reducing osteogenic function, inhibiting collagen, promoting osteoclast generation and prolonging its survival time, but also indirectly affects bone formation by regulating endocrine and related cytokines and inhibiting bone local blood flow (19). Therefore, unless it is necessary for treatment, we suggest that patients with osteoporosis should not use glucocorticoid as much as possible. With the increase of age, the secretion level of related hormones involved in bone metabolism is unbalanced, and the cytokines regulating bone metabolism decrease, resulting in an increase in bone loss. Therefore, we should pay more attention to the older adult patients with osteoporosis to prevent the occurrence of fractures.

According to the influencing factors of OPF obtained by multivariate regression analysis, we establish a prediction model and transform the complicated function formula into a visual nomogram for clinical medical staff to use. Each risk factor in the nomogram prediction model can get its corresponding score in the integral line at the top of the graph, and then the total score can be obtained by accumulating and summing each corresponding score one by one. Finally, the occurrence probability or prediction probability of stress injury of critically ill patients can be obtained on the line segment at the bottom of the nomogram prediction model, and the risk of injury can be obtained. If the data of osteoporosis patients are known, the predicted probability of OPF in this patient can be known. If a 40-year-old patient with osteoporosis has abnormal BMI and long-term use of glucocorticoids, BMD of lumbar, BMD of femoral neck and BMD of total hip are all abnormal values. The total score according to nomogram is 24.5, and the risk of OPF is 80%. Therefore, preventive measures should be taken in time in nursing to reduce the occurrence of fractures. The cutoff value of this model is 0.373, so the patients with *p* ≥ 0.373 are high-risk people of OPF, and vice versa. When the model is at the best cutoff value, the model has better sensitivity, better ability to identify patients with OPF at an early stage, and low probability of missed diagnosis. The model constructed in this study is developed based on the clinical data of real-world China population, which has important clinical significance for identifying the subgroup of osteoporosis patients with relatively high fracture risk.

Any prediction model is based on the epidemiological data of the national population, and the coefficients in the tool should be calibrated according to the demographic characteristics of the target population before use, so as to improve the prediction performance. The FRAX introduced by Britain is the most widely used, but it does not include the population in China (20). Korean Fracture Risk Score (KFRS) introduced by Korean scholars (21) is based on the national health database of Korea, and it is the first risk prediction model of individual OPF developed based on the Asian cohort, which can be used for risk screening in primary health care institutions lacking BMD testing equipment, but this tool is only suitable for Korean people. At present, the research on the risk of OPF in China is still in its infancy, and there is no risk prediction model based on OPF data of China population. The sample population of OPF risk prediction model constructed in this study is from China, which contains a variety of clinical risk factors, and it is simple and easy to use. It can be used in primary health care departments that lack bone mineral density testing equipment. The prediction model can be used to complete the initial screening of high-risk groups, and further testing can be carried out for the high-risk groups to confirm the diagnosis, so as to reduce medical costs and rationally allocate medical resources, and achieve a better cost–benefit ratio.

Although the construction effect of the prediction model is satisfactory, there are still some defects. First of all, because this study is retrospective, it is impossible to determine the causal relationship between fracture and some related factors. Secondly, the samples of this study are only from two hospitals, and its application in the country or internationally needs more external verification of central research. In addition, the new model classifies the risk factors into two categories, and does not further analyze such as repeated falls and multiple fractures. In the future, we hope to verify this prediction model in more external samples, and try to develop more risk prediction models of OPF with clinical value by using the independent influencing factors of OPF identified in this study.

## Conclusion

Through the analysis of risk factors, a fracture risk prediction model is established, which can be used to screen high-risk groups of fractures. At the same time, medical staff can evaluate the risk of patients by obtaining the medical history, and carry out hierarchical management intervention according to the evaluation results, and give effective prevention and treatment, which can achieve a better cost–benefit ratio.

## Data availability statement

The original contributions presented in the study are included in the article/supplementary material, further inquiries can be directed to the corresponding author.

## Ethics statement

The studies involving humans were approved by the Medical Ethics Committee of the First Affiliated Hospital of Soochow University (No. 2023-399) Suzhou China. The studies were conducted in accordance with the local legislation and institutional requirements. Written informed consent to participate in this study was not required from the participants in accordance with the national legislation and the institutional requirements.

## Author contributions

PX: Writing – original draft, Writing – review & editing, Funding acquisition, Investigation. YJ: Conceptualization, Data curation, Investigation, Methodology, Writing – review & editing. FC: Data curation, Investigation, Resources, Supervision, Writing – review & editing. SP: Data curation, Investigation, Visualization, Writing – review & editing. ZX: Conceptualization, Data curation, Formal analysis, Funding acquisition, Investigation, Project administration, Resources, Supervision, Writing – review & editing.
